# Menopause-Related Changes in Sleep and the Associations with Cardiometabolic Health: A Narrative Review

**DOI:** 10.3390/healthcare13172085

**Published:** 2025-08-22

**Authors:** Joshua R. Sparks, Xuewen Wang

**Affiliations:** 1 Independent Researcher, Columbia, SC 29208, USA; joshuasparks74@gmail.com; 2 Department of Exercise Science, Arnold School of Public Health, University of South Carolina, Columbia, SC 29208, USA

**Keywords:** menopausal transition, sleep disturbance, cardiometabolic risk, body composition, lipids, vascular health

## Abstract

This narrative review examines the complex relationship between sleep changes during the menopausal transition and cardiometabolic risks. The most common complaint about sleep is increased awakenings during sleep. Other complaints include having trouble falling asleep, waking up too early, insufficient and non-restorative sleep, and overall poor quality. Sleep determined using objective methods also indicates that greater awakenings after sleep onset are associated with the period of menopausal transition. Polysomnography recordings suggest physiological hyperarousal during sleep. Changes in other sleep metrics, such as sleep latency and sleep duration, are less consistent, and some studies suggest they may not worsen during the menopausal transition. These sleep issues are influenced by multiple factors, such as hormonal fluctuations, vasomotor symptoms, and psychosocial factors, and evidence suggests that hypothalamic kisspeptin/neurokinin B/dynorphin (KNDy) neurons are key underlying mechanisms for these associations. The menopausal transition is also associated with increases in cardiometabolic risk factors, such as body fat, altered lipid profiles, blood pressure, and vascular health. Emerging evidence suggests that poor sleep health during this period is associated with increased cardiometabolic risks and adverse cardiovascular outcomes. Thus, addressing sleep disturbances is crucial for comprehensive healthcare during the menopausal transition to safeguard long-term cardiometabolic health. Future research is needed to investigate interventions that can improve sleep and their impact on cardiometabolic health in this population experiencing increases in cardiometabolic risk.

## 1. Introduction

Menopause is retrospectively defined as the absence of menstrual bleeding in the past 12 months, a year after the final menstrual period (FMP), either naturally or due to medical reasons. The median age of natural menopause is 51–52 years, with a 95% confidence interval of approximately 45 to 55 years [1,2]. The menopausal transition generally starts a few years before the FMP and is usually noticed by women experiencing changes in menstrual cycle patterns. According to the Stages of Reproductive Aging Workshop (STRAW) + 10 staging system [3], the perimenopausal stage typically refers to the period from the start of menstrual cycle changes to a year after the FMP. The menopausal transition heralds the aging of the reproductive system and marks a significant physiological shift in a woman’s life. While the transition from premenopausal to postmenopausal represents a biological process, its impact can be profound, affecting various aspects of a woman’s life, including their physical health, emotional well-being, and interpersonal relationships [4,5,6].

It has long been known that the incidence of cardiovascular diseases notably increases after menopause [7]. In recent years, more detailed information regarding the changes in cardiometabolic risk factors during the menopausal transition has been published. These data demonstrate that there are accelerated increases in several cardiometabolic risk factors during the menopausal transition, and that these changes slow down a few years after the FMP [8]. Some examples of these factors include body fat mass, visceral fat, lean mass, and endothelial function [9,10,11,12,13]. Thus, slowing the increases in these risk factors during the menopausal transition may reduce women’s overall risk for future cardiovascular diseases.

The menopausal transition is often accompanied by some symptoms. A common and bothersome symptom concerns sleep, and nearly half of women reported waking several times a night three or more times a week [14]. Vasomotor symptoms such as hot flashes and night sweats can cause wakeups during sleep, although women who do not have vasomotor symptoms can also have worse sleep during the menopausal transition, suggesting that other causes also influence sleep [14]. Sleep is a complex physiological and behavioral process essential for maintaining psychological well-being and optimizing function across multiple physiological systems. The American Heart Association added sleep health to “Life’s Essential 8”, a construct of cardiovascular health, in 2022 [15].

The aims of this narrative review are to describe the characteristics of sleep changes during the menopausal transition and explore potential mechanisms for the changes in sleep characteristics and their contributions to cardiometabolic risk. In this narrative review, we will first detail the current literature on sleep characteristics during the menopausal transition and early postmenopausal period and discuss the potential mechanisms underlying the changes in sleep characteristics. Second, we will describe the changes in cardiometabolic risks during the menopausal transition. Third, we will explore the associations between sleep, cardiometabolic risk, and disease outcomes. We focus on sleep characteristics in those who do not have diagnosed sleep disorders. Lastly, we will discuss targeting improving sleep for cardiometabolic health.

This narrative review synthesizes the existing literature on sleep characteristics during the menopausal transition, with a focus on individuals without diagnosed sleep disorders, providing a targeted exploration linking sleep changes with cardiometabolic risk. By focusing on this specific population and emphasizing the potential for targeting sleep to improve cardiometabolic health, this review offers novel insights not readily available in analyses of menopause-related sleep disturbances or cardiometabolic risk alone.

## 2. Sleep During the Menopausal Transition and Postmenopausal Period

In this section, we structure our discussion of sleep characteristics using self-reported and objectively measured methods and then discuss factors linked to menopause-related sleep disturbances. Given the volume of research on this topic, we examined reviews and meta-analyses and included the synthesized information. For individual studies, we prioritized findings from large-scale studies and/or studies reporting longitudinal changes across menopausal transition. Our discussion of factors associated with sleep disturbances includes both well-established factors and recent discoveries.

### 2.1. Self-Reported Sleep Disturbances

Self-reported sleep disturbances during the menopausal transition are characterized by increased awakenings during sleep and can also include having trouble falling asleep, waking up too early, insufficient and non-restorative sleep, and overall poor quality. Waking several times per night has the highest prevalence compared to early morning wakening and trouble falling asleep among all sleep-related complaints in women across the menopausal transition [14]. A substantial number of women experience sleep disturbances during the menopausal transition. A recent review summarized findings from studies in several countries and continents [16]. The authors found that the prevalence of sleep disturbance associated with menopause was 43–69%, and the proportion of women meeting the criteria for an insomnia diagnosis was between 4% and 40%.

Sleep disturbances become more pronounced when women enter the menopausal transition. The annual National Survey of Health and Development in the United Kingdom (UK) revealed that, in women aged between 48 and 54, severe sleep difficulty increased approximately 2- to 3.5-fold for women in menopausal transition compared with women who remained premenopausal [17]. In the Study of Women’s Health Across the Nation (SWAN), a national study in the United States (US), the prevalence of difficulty sleeping, including trouble falling asleep, waking several times throughout the night, and waking up earlier than intended, increased linearly from the premenopausal period (30%) to the early and late perimenopausal periods (early: 40%; late: 45%), and plateaued from the late perimenopausal period into the postmenopausal period (40–45%) [18]. In Korean women, peri- and postmenopausal women were 1.50 and 1.73 times more likely to have poor sleep quality in comparison to premenopausal women [19]. In addition, data suggest that more than half (56.0%) of women during the perimenopausal period are more likely to sleep less than 7 h on average in a 24 h period, compared with 32.5% of premenopausal and 40.5% of postmenopausal women [20]. These data indicate that the prevalence of self-reported sleep disturbances increases with the onset of menopausal transition, plateaus a few years after the FMP, and may slightly improve afterwards.

However, individuals may present different patterns of change over the menopausal transition. The SWAN study found four distinct trajectories across the menopausal transition (from -10 years to +10 years of the FMP) for waking several times per night in 1285 women: low prevalence (37.9%), moderate prevalence (28.4%), increasing prevalence (15.3%), and high prevalence (18.4%) [14]. Individuals who follow the trajectories of increasing prevalence and high prevalence need particular attention; however, the menopausal transition may play a more significant role for those who experience increased prevalence during the menopausal transition.

### 2.2. Objectively Measured Sleep Metrics

Several studies have used objective methods to measure sleep during the menopausal transition. As with self-reported sleep disturbances, studies objectively measuring sleep also found that waking after the onset of sleep was more common than difficulty falling asleep (longer sleep onset latency) in women during the menopausal transition [21]. As part of a larger prospective “Woman 46” study, premenopausal women aged 46 years were followed for 6 and 10 years; 60 had complete data at a 6-year follow-up visit [22], and 57 had complete data at a 10-year follow-up visit [23]. Women were in different stages of the menopausal transition at the 6-year follow-up visit. On average, they had a shorter total sleep time and lower sleep efficiency, as well as increased transitions from slow-wave sleep to wakefulness, waking after sleep onset, awakenings per hour, arousal index, and apnea–hypopnea index than at baseline [22]. At the 10-year follow-up visit, all women were postmenopausal. Compared to the baseline, when they were premenopausal, they had a shorter sleep latency, a shorter latency to stage 2 sleep, decreased stage 2 sleep, increased slow-wave sleep, an increased number of awakenings and arousals per hour, and a greater arousal index and apnea-hypopnea index [23]. The authors noted that there was not a significant change from the 6-year to 10-year examinations, suggesting that alterations to sleep parameters are predominantly due to the menopausal transition and plateau as women enter the postmenopausal period of their lives. However, these two studies are limited due to small sample sizes.

A larger study of 300 women in the SWAN study had at least two out of three sleep assessments (mean age of 52 years at the baseline and follow-ups after 3 and 12 years) [24]. Their sleep duration increased over time. Sleep latency was similar at baseline and the 3-year follow-up but increased thereafter. Waking after sleep onset increased from baseline to 3 years, declining thereafter. The authors suggest that sleep may not worsen, in general, in midlife women. Another study compared 4- and 8-year follow-ups with the baseline (aged 30–60 years), with an adjustment for age [25]. They found that postmenopausal women had longer deep sleep (stages 3 and 4) and a longer total sleep time than premenopausal women, and perimenopausal women had significantly less light sleep (stage 1) and more deep sleep than premenopausal women. Sleep latency did not differ by menopausal status. However, self-reported dissatisfaction with sleep was approximately two-fold higher in perimenopausal and postmenopausal women than in premenopausal women. Interestingly, a small study found that peri- and late postmenopausal women had higher relative amplitude and stability in circadian rhythms than premenopausal women [26]. Collectively, more awakenings after sleep onset are associated with the period of menopausal transition and may improve afterwards. Other sleep metrics, such as sleep latency and sleep duration (total and specific sleep stages), may not become worse during the menopausal transition.

Polysomnography provides additional information. In a cross-sectional study, data revealed elevated beta electroencephalogram (EEG) power in the rapid eye movement (REM) and non-rapid eye movement (NREM) phases of sleep in late perimenopausal and postmenopausal women compared to pre- and early perimenopausal women [27]. In a longitudinal study, women who transitioned to the postmenopausal period had higher beta NREM EEG power, compared to women who remained pre- or early premenopausal, at the follow-up time [28]. The elevated beta EEG power suggests that the menopausal transition is associated with increased arousal levels during sleep, which seems to be more pronounced during the NREM stage. These data, again, suggest that the menopausal transition is associated with physiological hyperarousal during sleep [28].

### 2.3. Factors Linked to Sleep Disturbances During the Menopausal Transition and Postmenopausal Period

Many factors are known to influence sleep. Hormonal changes, vasomotor symptoms, and psychosocial factors are important contributors to menopause-related sleep disturbances. Growing knowledge suggests that neurons that integrate the hormonal regulation of sex hormones and the circadian regulation of sleep–wake cycles may be a key mechanism.

The link between sex hormone fluctuations and sleep is not limited to the menopausal transition. In cyclic women, poorer sleep quality is common before and during the menstrual phase in some women [29]. Although objective sleep continuity may not change across the menstrual cycle, a prominent increase in sleep spindle activity in EEGs has been observed in the luteal phase when progesterone levels are relatively high compared to the follicular phase [30]. During the menopausal transition, the fluctuations of estrogen and follicle-stimulating hormone (FSH) last several years, which correspond to the time when sleep disturbance deteriorates. In addition, hormone replacement therapy (HRT), both estrogen alone and estrogen combined with progestin, is effective in reducing sleep disturbances throughout the menopausal transition and early postmenopausal period to some extent, mostly regarding self-reported sleep [31,32]. However, the link between sex hormones and sleep disturbance may be complicated as some studies do not observe a significant association. For example, in the SWAN study, no differences in FSH or estrogen concentrations were found among the different trajectories of sleep disturbance over the menopausal transition as discussed earlier [14].

The benefits of HRT on sleep may be related to the alleviation of vasomotor symptoms such as hot flashes and night sweats, which can disrupt sleep [33]. More than half of women experience frequent vasomotor symptoms during the menopausal transition, with symptoms persisting for 4 to 7 years [34,35,36]. Studies have shown that women experiencing more severe vasomotor symptoms are more likely to report greater sleep disturbances, poorer sleep quality, and impaired daytime functioning [37,38,39,40]. Hot flashes were also found to be associated with polysomnography alterations suggestive of insomnia [41]. Night-time vasomotor symptoms were associated with increased waking after sleep onset using actigraphy and polysomnography [42,43]. Another study found that actigraphy-assessed waking episodes were concurrent with 78% of objectively determined hot flashes, irrespective of whether women reported the hot flash [44]. Vasomotor symptoms can last several years, highlighting their potential ongoing impact on sleep. Despite concerns of effects on other health outcomes, HRT remains the primary treatment for bothersome vasomotor symptoms [45]. Therefore, careful consideration is warranted for managing vasomotor symptoms and sleep [46].

Mood symptoms, most notably mood swings and depressive symptoms, are also common during the menopausal transition [47,48]. The relationship between mood symptoms and sleep disturbance is likely bidirectional [16]. Difficulty in sleeping largely explained the relationship between vasomotor symptoms and subsequent depressed mood [49], and an improvement in sleep predicted an improvement in depression [50]. Higher depressive symptoms were associated longitudinally with actigraphy-measured poorer sleep health in midlife women in the SWAN study [51]. During the menopausal transition, the relationships among vasomotor symptoms, mood symptoms, and sleep disturbances are intricate, and some suggest that the order of symptom occurrence may affect these relationships [49]. Additionally, alterations in circadian rhythm may occur and contribute to menopause-related sleep disturbances [52,53]. Social determinants of health, such as the demands of caregiving and the constraints imposed by socioeconomic status, may influence sleep through time constraints, stress, and other pathways [54], which may be pronounced in this population.

Recent evidence suggests that neurons that integrate the hormonal regulation of sex hormones and the circadian regulation of sleep–wake cycles may be a key underlying mechanism linking changes in sex hormones and sleep, as well as vasomotor and mood symptoms during the menopausal transition [16]. Hypothalamic kisspeptin/neurokinin B/dynorphin (KNDy) neurons express neuromodulators of the gonadotropin-releasing hormone (GnRH) pathway, which regulates the secretion of sex hormones. KNDy neurons also take part in the homeostatic control of body temperature. A neurokinin-1,3 receptor antagonist (KNDy neurons express the neurokinin-3 receptor) improves sleep and mood outcomes in postmenopausal women [55,56]. Many of the regulatory mechanisms are yet to be characterized and understood.

## 3. Changes in Cardiometabolic Risks During the Menopausal Transition

There is a notable increase in the incidence of cardiovascular diseases in women during midlife, which coincides with the period of the menopausal transition and has been observed for at least a few decades [7]. The American Heart Association published a scientific statement discussing menopause and cardiovascular risk in 2020 [8]. Therefore, we leverage this publication and summarize the existing literature with additional information that has become available in the last five years.

### 3.1. Body Composition and Fat Distribution

During the menopausal transition, on average, the amount of body fat increases [57], and the body mass index (BMI) does not sufficiently reflect adiposity in postmenopausal women, in that women with a normal BMI can have a high body fat percentage [58]. Longitudinal data from the SWAN study have shown that changes in fat mass and lean mass during the menopausal transition are distinct from before and after this period. Specifically, fat mass and lean mass increase prior to the menopausal transition [9]. From 2 years before to 2 years after the FMP, the rate of fat mass gain doubles, and lean mass declines. After this, the changes in fat mass and lean mass decelerate and become stable. Bone loss also accelerates during the period of menopausal transition [59,60]. The decreases in lean mass and bone mass with a simultaneous increase in fat mass reduce the degree of weight gain related to menopause. In fact, weight gain in midlife was found to be largely a product of chronological aging [61,62,63,64].

Other than an increase in total fat mass, accelerated increases in abdominal fat and visceral adipose tissue also occur [57,65,66,67]. While visceral adipose tissue does not significantly change in the 2 or 3 years before the FMP, it has been reported to increase by approximately 6–8% per year during the menopausal transition [10,11], and to decelerate to a 1.47% per year increase after the transition [10]. Android fat (fat in the android region as determined by dual-energy X-ray absorptiometry) followed a similar trajectory, increasing by 5.54% during the menopausal transition [11]. These changes were independent of age at the FMP and HRT. The increase in visceral adipose tissue from 2 years before the FMP to the FMP has been shown to be associated with a significant increase in subclinical atherosclerosis [10], demonstrating the association between adiposity and the development of cardiovascular disease during the menopausal transition.

### 3.2. Lipids and Glucose

Changes in lipids, glucose, and insulin also occur during the menopausal transition [8,62]. Recent data have shown complex changes, especially for high-density lipoprotein cholesterol (HDL-C). An analysis of data from the SWAN study indicates that there are two trajectories each for low-density lipoprotein cholesterol (LDL-C), apolipoprotein B, HDL-C, triglycerides (log-transformed), and apolipoprotein A-I across the menopausal transition [68]. One trajectory shows an increasing trend before menopause but a decreasing trend afterwards (inverse U-shape), distinct from the other trajectory, which is relatively slow-progressing or stable, therefore indicating the influence of menopause. Interestingly, the quality of the functional capacity of HDL changes during the menopausal transition [69], resulting in a reversal in the direction of the association between HDL-C and carotid atherosclerosis, with higher HDL-C levels associated with less carotid atherosclerosis before menopause but with greater carotid atherosclerosis after menopause [70]. A cohort of Chinese women showed that total cholesterol, LDL-C, and triglycerides began to increase in early transition, and the maximum annual increase was from 1 year before to 2 years after the FMP for total cholesterol and LDL-C, and from 1 year before to 4 years after the FMP for triglycerides [71]. The changes in HDL-C, however, appeared to differ depending on the age at baseline observation and BMI.

The changes in glucose metabolism after menopause are suggested to be primarily due to chronological aging and changes in body composition, not the menopausal transition per se [62,72,73]. However, studies that have assessed insulin secretion and insulin sensitivity, two key aspects of glycemic control, across menopausal transition are scarce. A recent community-based cohort study tracked β-cell function and insulin sensitivity over time in the period approximating menopause [74]. They found that women with different timelines of diabetes onset (before, during, and after the menopausal transition) all exhibited lower β-cell function and insulin sensitivity during the period of menopausal transition compared with women who did not develop diabetes. However, women who developed diabetes in the perimenopausal period showed decreases with fluctuations, while those who developed diabetes in the postmenopausal period showed maintenance in their values. Interestingly, the study found that the odds ratio of developing diabetes gradually increased during the premenopausal period, was sustained during the menopausal transition, and decreased in the postmenopausal period.

### 3.3. Blood Pressure and Vascular Health

Two-thirds of women aged 60 years and older have hypertension [75]. Longitudinal data from the SWAN study found three trajectories of changes in blood pressure across menopause [76]. The most distinctive trajectory (35%, 53%, and 28% of the cohort for systolic blood pressure, pulse pressure, and mean arterial pressure, respectively) shows a slow increase before menopause and a significant accelerated rise one year after menopause, indicating the contribution of menopause. The other two trajectories increased until menopause and either continued with the same increase or declined after menopause. These findings suggest that women with low baseline cardiovascular risk had an acceleration of hypertension risk following the menopausal transition, in contrast with others who were at higher risk before menopause and did not experience the same degree of increase in blood pressure following menopause [77].

In a cross-sectional study of women with a wide age span (18–70 years), flow-mediated dilation declined with age, with a breakpoint and steeper decline occurring at 47 years of age, and the carotid-femoral pulse wave velocity was relatively stable until a breakpoint at age 48, increasing afterwards [12]. The ages of endothelial function decline and an increase in aortic stiffness correspond to the start of the menopausal transition. The study also found that FSH and progesterone partly explain the associations between age and endothelial dysfunction, suggesting the role of menopause. Longitudinal data from the SWAN study provided further details of the change in atrial stiffness across the menopausal transition, i.e., a significant 7.5% annual increase from FMP −1 year to +1 year [13].

## 4. Association of Sleep with Cardiometabolic Risk and Diseases

There is an increasing body of evidence demonstrating the importance of healthy sleep in maintaining cardiometabolic health. Insufficient sleep duration and poor sleep quality have been linked with increased cardiometabolic risk factors [78,79,80,81], subclinical [82,83,84] and cardiovascular diseases [85,86,87,88,89,90], and decreased life expectancy free of cardiovascular diseases [91]. Sleep architecture has also been linked with various health conditions, including cardiovascular risks and issues [92,93,94].

The associations between sleep and cardiometabolic diseases are bidirectional, and the mechanisms responsible for the associations are multifactorial. Sleep disturbances may influence behavioral factors, such as dietary intake and participation in physical activity, which subsequently affect cardiometabolic health; on the other hand, having cardiometabolic conditions may influence sleep [95,96,97]. Furthermore, cardiometabolic functions, such as endothelial function, blood pressure, glucose metabolism, and the stress response, are regulated by the circadian clock [98,99]. Sleep disturbance often disturbs the regularity of the circadian rhythm, which may impact cardiometabolic functions [100,101].

Fewer studies have examined the associations between sleep and cardiometabolic risk in women during the menopausal transition. In the SWAN study, four trajectories of self-reported insomnia symptoms over 22 years were found: low symptoms, moderate symptoms decreasing over time, low symptoms increasing over time, and high symptoms that persisted [102]. Women with persistently high insomnia symptoms had higher cardiovascular disease risks than those with low insomnia symptoms, and this association persisted after adjustment for vasomotor symptoms, snoring, or depression. A subgroup of the SWAN study found that poor self-reported sleep quality was associated with calcification in the aorta but not in the coronary arteries in healthy midlife women [103]. In a national cohort in China, individuals 45 years and older were followed for seven years [104]. Individuals with a good to poor changing pattern of sleep quality (number of restless sleep days in the past week changed from <3 to 3–7 days) or consistently poor sleep quality were associated with an increased risk of cardiovascular diseases compared to those with a consistently good longitudinal pattern, with adjustments for age, sex, BMI, and multiple additional covariates. Among Chinese women aged 45–55 years, self-reported sleep and physical activity have an additive association with dyslipidemia risk [105]. Women with sleep problems had higher dyslipidemia risk levels than women without sleep problems, irrespective of physical activity level. These studies suggest that sleep health during the menopausal transition is associated with cardiometabolic risk.

There is also evidence for the influence of menopausal status on the association between sleep and cardiometabolic health. In women aged 40–60 years who were free of cardiovascular diseases, poorer self-reported sleep quality was associated with elevated arterial stiffness, as measured by brachial ankle pulse wave velocity, in perimenopausal women but not in premenopausal women [106]. Another study examined the impact of mild sleep restriction lasting 6 weeks (reduction of 90 min per night from adequate sleep) and found that results varied by menopausal status [107]. Total cholesterol and LDL-C decreased following sleep restriction in premenopausal women but not in postmenopausal women. However, further studies are needed to further examine how menopausal status affects these associations and how the shifts in associations occur during the menopausal transition.

## 5. Targeting Sleep to Reduce Menopause-Related Increases in Cardiometabolic Risk

The evidence collectively supports an association between sleep and cardiometabolic health during the menopausal transition, although more investigations specific to this population are needed. Addressing sleep disturbances and promoting healthy sleep habits may help preserve metabolic health and reduce the risk of cardiometabolic diseases in women transitioning from the pre- to postmenopausal period.

A variety of interventions have been used to improve sleep. Cognitive behavioral therapy for insomnia (CBT-I), for instance, has been shown to be highly effective in treating insomnia symptoms [108]. CBT-I helps individuals address maladaptive sleep habits and thought patterns, leading to improved sleep quality and reduced sleep disturbances. Implementing mind–body exercises and relaxation techniques can also improve sleep quality and duration [109]. A systematic review of 15 randomized controlled trials concluded that CBT-I was most consistently associated with improved hemoglobin A1C and inflammatory marker C-reactive protein (CRP) levels [110]. The effects varied or were null for inflammatory markers such as interleukin-6 (IL-6) and tumor necrosis factor-α (TNF-α), as well as systolic and diastolic blood pressure. A few studies have examined the effects of extending sleep duration and have suggested beneficial effects on cardiometabolic risks, such as reductions in fasting insulin, glycemic variability (via continuous glucose monitoring), and improved insulin secretion and insulin resistance [111,112], as well as dietary choices [113,114]. However, the beneficial effects are not shown in other studies [115,116], suggesting that the results are influenced by an individual participant’s level of cardiometabolic risk, the amount of sleep extended, and the measurement method.

Few studies have been conducted to specifically examine the effects of sleep interventions on cardiometabolic health in women during the menopausal transition. Several approaches have been used to treat menopause-related sleep issues, such as nutritional interventions and psychological therapies, although results show significant heterogeneity [117,118,119,120]. Additionally, emerging evidence suggests a weakened circadian signal as women age and transition through menopause [121]. Thus, circadian and light therapy may be potential approaches to improving menopause-related sleep issues. More studies are being conducted to further characterize and understand the circadian alterations in women across the reproductive aging stages [122]. Importantly, whether these interventions for sleep can subsequently improve cardiometabolic health remains to be investigated.

The changes with interventions for sleep may occur directly or indirectly through psychological, behavioral, and physiological mechanisms [123]. Combining sleep management with lifestyle and behavioral management, such as increasing physical activity, promoting a heart-healthy diet, and stress management, will likely result in better overall cardiometabolic outcomes in these individuals. Comprehensive interventions should consider integrating individual-level behavioral modifications with strategies that address social determinants of health. High-quality studies in women experiencing menopause-related sleep disturbances are highly needed to determine the potential for targeting sleep to improve cardiometabolic health.

## 6. Conclusions

A substantial number of women experience sleep disturbances during the menopausal transition, and sleep disturbances become more pronounced when women enter the menopausal transition. Data from actigraphy indicate that more awakenings after sleep onset are associated with the period of menopausal transition and may improve afterwards. Other sleep metrics, such as sleep latency and sleep duration (total and in specific sleep stages), may not worsen during the menopausal transition. However, individuals may present with different patterns of change. Polysomnography shows more pronounced elevations of beta EEG power during the NREM stage than during other stages during the menopausal transition. Many factors may contribute to menopause-related sleep disturbances. The menopausal transition is also associated with increased cardiometabolic risks, such as changes in body composition, body fat, blood lipids, glucose, blood pressure, and vascular health. The current evidence supports an association between sleep and cardiometabolic health during the menopausal transition, although more investigations specific to this population are needed. Addressing sleep disturbances and promoting healthy sleep may help reduce the risk of cardiometabolic diseases in women transitioning from the pre- to postmenopausal period. Future studies are greatly needed to examine whether targeting sleep can benefit cardiometabolic health in women transitioning through menopause.

Importantly, as a narrative review, this work is subject to limitations, including the potential biases of the authors, the incomplete retrieval of all relevant studies, and the subjective selection of the included research. These factors could influence the comprehensiveness and accuracy of the discussion. Nonetheless, we provide a focused review of sleep changes during the menopausal transition and explore their relationship with increased cardiometabolic risk during this period, laying the groundwork for future research advancing awareness of menopause-related changes in sleep and addressing sleep issues for its potential effects on cardiometabolic health.
